# Report of a Case of Croupous Dysentery

**Published:** 1897-01

**Authors:** G. O. Jones

**Affiliations:** Stone Mountain; Lithonia, Ga.


### REPORT OF A CASE OF CROUPOUS DYSENTERY.

G. O. JONES, M. D., STONE MOUNTAIN, GA.

I was called in consultation by a fellow practitioner on December 8th, to see a patient who had been suffering for over nine weeks—with what was supposed to be catarrh of the bowels.

The physician in charge said the patient was first taken sick very suddenly with bloody discharges at the onset.

He had given everything he could think of to check the discharges, which continued in spite of the most heroic astringent treatment.

On examination, I found some tenderness over the right iliac fossa, with an induration plainly outlined.

My attention was then called by the physician to a protrusion from the anus which at first appeared to be a portion of the lower gut proper, but on examining it carefully, it proved to be the mucous lining of the gut.

This lining was the exact size of the original gut and was entire with the exception of a few small perforations. Finding no vitality in the membrane I clipped it off, as it annoyed the patient.

On the following morning I visited the patient again and found that just previous to my visit he passed eighteen inches more of the lining membrane.

The patient by this time had become very weak and was almost overcome by the exertion of passing this membrane. I ordered stimulants and nourishment, gave strychnine sulphate one-thirtieth grain, and liquid peptonoids.

Up to this time he had taken but little stimulants. Sent for Dr. Kendrick, of Atlanta, to consult with me. He arrived the following day. Patient by this time had grown so weak by constant drainage from the bowels consisting mostly of blood, that under no stimulant did he rally and died the following morning, after having had fifteen passages during the night.

I report this case as it is one not met with very often.

---

### NEW METHOD FOR REDUCING HERNIA.

Stafford, in *American Medico Surgical Bulletin*, advises continuous pressure for reducing a hernia. An ordinary rubber bandage, 2½ inches wide and 3 yards long, is wound about the scrotum and penis, commencing below the centre and drawing tighter at the lowest part, until all the parts is covered. This is less painful and more effective than taxis. The same method is employed for prolapsed rectum.

